# Two reassortant types of highly pathogenic H5N8 avian influenza virus from wild birds in Central China in 2016

**DOI:** 10.1038/s41426-017-0012-y

**Published:** 2018-02-07

**Authors:** Liping Ma, Tao Jin, Hanzhong Wang, Haizhou Liu, Runkun Wang, Yong Li, Guoxiang Yang, Yanping Xiong, Jing Chen, Jun Zhang, Guang Chen, Wei Li, Di Liu, Peng Lin, Yueying Huang, George F. Gao, Quanjiao Chen

**Affiliations:** 1 CAS Key Laboratory of Special Pathogens and Biosafety, Wuhan Institute of Virology, Chinese Academy of Sciences, Wuhan, 430071 Hubei China; 2 Center for Influenza Research and Early-Warning (CASCIRE), Chinese Academy of Sciences, Beijing, 100101 China; 3 University of Chinese Academy of Sciences, Beijing, 100049 China; 4 China National Genebank-Shenzhen, BGI-Shenzhen, Shenzhen, 518120 China; 5 The monitoring center of wildlife diseases and resource of Hubei, Wuhan, 430071 China; 6 Institute of Microbiology, Chinese Academy of Sciences, Beijing, 100101 China; 7 Office of Director-General, ChineseCenter for Disease Control and Prevention (China CDC), Beijing, 102206 China

## Abstract

Since 2016, the highly pathogenic avian influenza H5N8 virus has emerged in the Central Asian flyway and Europe, causing massive deaths in poultry and wild birds. In this study, we isolated and identified three H5N8 viruses from swan goose and black swans in Hubei province during the 2016/2017 winter season. Whole-genome sequencing and phylogenetic analysis revealed that the three viruses clustered into a group of H5N8 viruses from Qinghai Lake and Europe. A novel reassortment virus from swan goose was distinguished from that of black swans, in that its *PA* and *NP* genes were distinct from those of Qinghai Lake viruses. Molecular dating revealed that the ancestral strain of these H5N8 viruses emerged around July 2015. From sequence comparison, we discovered eight amino acid substitutions in HA and NA during the adaption process from poultry to wild birds. The three viruses were isolated from wild birds in the East Asian-Australasian flyway; however, the viral genomes were similar to H5N8 viruses circulating along the Central Asian flyway. From these data, we conclude that wetlands and lakes in Central China may play a key role in disseminating H5N8 viruses between the East Asian-Australasian and Central Asian flyways.

## Introduction

Early in 2014, highly pathogenic avian influenza (HPAI) H5N8 viruses, belonging to clade 2.3.4.4, were monitored in wild birds and poultry in nine European and Asian countries^1^. In late 2014, the virus emerged in North America, then in domestic and wild birds until mid 2015^2^. In 2015, H5N8 viruses were isolated in China, Taiwan, Hungary, and Sweden in wild birds and poultry^1^. In 2016, a novel lineage emerged in wild birds in Qinghai Lake, spread to Mongolia, Siberia, and Europe, and was identified as clade 2.3.4.4 Group B. The 2014/2015 viruses were categorised into clade 2.3.4.4 Group A^3^. This reassortment H5N8 virus led to epidemics in wild birds in 40 countries, with most outbreaks observed in Europe (28 countries in Europe, 4 in Asia, 3 in the Middle East, and 5 in Africa)^4^. H5N8 virus has become widespread among wild birds worldwide.

Before December 2016, H5N8 had disseminated ~22 times in China, including domestic and wild birds^4–10^. Since the first H5N8 isolate (A/duck/Jiangsu/k1203/2010) was detected in Jiangsu province, two different reassortants appeared in poultry during 2012–2014 in China^6, 10^. H5N8 detection rateis extremely low in live poultry markets (LPMs) in China, and no H5N8 virus was isolated in our routine sampling from 2015 to 2017. Moreover, the viruses circulating in domestic poultry in 2011–2014 in China disappeared after 2015. However, novel H5N8 ressortants have been observed in wild and domestic birds in Europe (over 3000 outbreaks) since 2016.

H5N8 outbreaks usually occur in winter (November–February), consistent with the migration time of wild birds, suggesting that the spread of H5N8 throughout the world is closely associated with bird migration. However, because of our limited knowledge of HPAI in wild birds, the roles of these wild birds in disseminating the virus and the mechanisms through which the virus spreads among different flyways are unclear^11^.

Accordingly, in this study, we performed surveillance of avian influenza virus in LPMs and migratory birds in Central China monthly from November 2016 to March 2017, during the period in which migratory birds fly to wetlands and lakes for overwintering. In addition, we explored the phylogenetic relationships and amino acid mutations in H5N8 virus interspecies transmission from poultry to wild birds.

## Materials and methods

### Ethics statement

All studies involving animals were conducted according to the animal welfare guidelines of the World Organisation for Animal Health. Fresh migratory bird faeces were collected in Wang Lake Wetland Reserve (longitude: 115.22°E, latitude: 29.83°N) and other lakes in Hubei province with specific permission from the Monitoring Center of Wildlife Diseases and Resource of Hubei Province, China. The field studies did not involve endangered or protected species. The cloacal swabs were collected in LPMs in Central China and no specific permissions were required for these activities.

### Sample collection and virus isolation

Sterile cotton swabs were used to collect fresh migratory bird faeces and cloacal swabs in LPMs. The swabs were placed into 1 mL viral transport medium, transported to the laboratory within 24 h at 4 °C, and then frozen at −80 °C. Swabs and viscera tissues, such as the rectum and lungs, were also obtained from dead Anser cygnoides from the Wang Lake Wetland Reserve and dead Cygnus atratus from a zoo in Wuhan city, China. Viruses were isolated from 10-day-old specific pathogen-free (SPF) chicken embryos or Madin-Dardy canine kidney (MDCK) cells according to the World Health Organisation (WHO) manual (WHO, 2002). Hemagglutinin (HA)-positive samples were further confirmed by reverse transcription PCR (RT–PCR) using universal primers targeting the *M* gene^12^.

### RNA extraction, RT–PCR, and detection

HA-positive allantoic fluid and cell culture supernatants of infected cells were collected for RNA extraction using a Nucleic Acid Extraction System with matched EX-RNA/DNA viral nucleic acid extraction kits (Tianlong Science and Technology, Co., Ltd.). cDNAs were synthesised from vRNAs by reverse transcription with Uni12 and Uni13 primers^12^. Subtype identification was carried out as described previously^12^.

### Sequencing

Next-generation sequencing (NGS) was used to determine the whole-genome sequences of three avian influenza virus isolates. Briefly, the sequencing libraries were prepared and sequenced on an Illumina HiSeq 4000 Sequencer as described previously^13^. For three viruses, the whole-genome sequences were submitted to GenBank database (accession numbers: MF040669–MF040692).

### Sequencing data filter and assembly

The raw reads were processed by filtering out low-quality reads (10 bases with qualities <10), adaptor-contaminated reads (with N15 bp matched to the adapter sequence), poly-Ns (with 10 Ns), duplication, and host-contaminated reads (SOAP; <5 mismatches). The filtered reads were mapped to the INFLUENZA database to choose the best-matched reference sequences. We then used MAQ to perform reference-based assembly^14^. In addition, the remaining filtered reads were subjected to de novo assembly using SOAPdenovo (version 1.06)^15^ and edena (v3.121122), respectively. The de novo contigs (>200 bp) were aligned to the reference-based assembly sequences, to correct for indels and mismatches. The second running of MAQ was performed to generate the final sequences based on the improved sequences obtained from the combination of the above-mentioned three assemblies.

### Phylogenetic analyses

Maximum-likelihood phylogenetic analysis for *HA* (1704 nucleotides) and *PA* (2151 nucleotides) genes of H5N8 influenza viruses, from the HPAI H5N8 viruses circulating in China in 2010–2016, newly released H5N8 strains in 2016–2017 available from Global Initiative on Sharing All Influenza Data (GISAID, http://platform.gisaid.org/epi3/frontend), and the top BLAST hits for the segments was performed with RAxML-HPC2 on the Extreme Science and Engineering Discovery Environment (XSEDE) version 8.2.10^16^ with 1000 bootstraps. Each tree was rooted with the earliest isolate among the selected strains.

Maximum clade credibility (MCC) trees of *HA* (1704 nucleotides) and *NA* (1395 nucleotides) genes were inferred by Bayesian Evolutionary Analysis using Sampling Trees (BEAST) v1.8.3 under the HKY substitution model, with a strict clock and chain length of 50,000,000. The dating sequences were the top BLAST hits of the first H5N8 isolate (A/duck/Jiangsu/k1203/2010) and the three H5N8 strains in this study. Moreover, Tracer v1.6 was used to confirm the reliability of the results. All trees were summarised by Tree Annotator with 10% burn-in cutoffs, and the maximum clade credibility trees were visualised and decorated by FigTree v1.4.3.

### Amino acid host adaptation analysis

We collected HA and NA amino acid sequences of H5N8 viruses and divided the sequences into three groups: Group A, 2010–2014 domestic ducks in East Asian-Australasian flyway (91 isolates, 91 HA amino acid sequences, and 49 NA amino acid sequences); Group B, 2010–2014 wild birds (70 isolates, 70 HA amino acid sequences, and 53 NA amino acid sequences) in the East Asian-Australasian flyway; and Group C, 2016–2017 wild birds (101 isolates, 101 HA amino acid sequences, and 95 NA amino acid sequences) in the Central Asian flyway and Europe. Amino acid sequences were aligned by BioEdit Sequence Alignment Editor, and the sequence variation was analysed and visualised by WebLogo^17^.

## Results

### Virus detection, isolation, and subtyping

Since 2015, we have been conducting surveillance of LPMs and wetlands and lakes in Central China (Hubei, Hunan, and Jiangxi provinces) and collected ~2400 samples from LPMs and 4000 samples from wild birds each year. Notably, no H5N8-positive samples were detected in LPMs. In particular, from a total of 1977 samples from wild birds during November 2016 and March 2017, three H5N8 viruses were isolated, one (A/Anser cygnoides/Hubei/FW44/2016, FW44) from a dead swan goose in the Wang Lake Wetland Reserve (longitude: 115.22°E, latitude: 29.83°N) on December 13 during routinesampling and the other two (A/Cygnus atratus/Hubei/2Z2-O/2016, 2Z2-O and A/Cygnus atratus/Hubei/HF-1/2016, HF-1) from dead black swans (Cygnus atratus) in a zoo (longitude: 114.24°E, latitude: 30.54°N) in Wuhan on December 20 and 28syndromic sampling, respectively. Notably, the black swans live in an island of the zoo, which is located in the center of habitat of black swan, where gathered many migratory birds, and the black swans can interact with wild migratory birds freely. The H5N8 infection in the zoo was also confirmed by the National Avian Influenza Reference Laboratory (NAIRL). Since January 2017, a total of 99 swans died in zoo, and 215 were culled^18^. In addition, the zoo was located 129.6 km away from the Wang Lake Wetland Reserve, and migratory wild birds usually stop over in these two places.

### Sequence and phylogenetic analysis

The two swan isolates shared 99.2–100% homology. The virus from the swan goose shared 98.5–99.5% homology with the swan isolates, except for the PA and NP genes, which showed lower homology (95.8% and 97.3–97.4%, respectively). All genes from the three isolates shared high homology to those of the 2016–2017 H5N8 viruses isolated worldwide (Table 1).

**Table 1:** The highest nucleotide similarity of the two H5N8 viruses in this study, with the sequences from global initiative on sharing all influenza data (GISAID)

Isolate name	Gene	Viruses with the highest nucleotide identity (%)	Accession number	Homology (%)
A/Anser cygnoides/Hubei/FW44/2016(H5N8)	*PB2*	A/painted stork/India/10CA03/2016 (H5N8)	EPI858841	99.7
	*PB1*	A/great crested grebe/Uvs-Nuur Lake/341/2016 (H5N8)	EPI773755	99.5
	*PA*	A/painted stork/India/10CA03/2016 (H5N8)	EPI858843	99.6
	*HA*	A/turkey/Poland/83/2016 (H5N8)	EPI869931	99.6
	*NP*	A/domestic duck/Siberia/103/2016 (H5N8)	EPI926614	99.8
	*NA*	A/Brown-headed Gull/Qinghai/ZTO6-MU/2016 (H5N8)	EPI774500	99.4
	*MP*	A/mallard duck/Korea/WA137/2017 (H5N8)	EPI952800	99.4
	*NS*	A/domestic duck/Siberia/103/2016 (H5N8)	EPI926617	99.6
A/Cygnus atratus/Hubei/HF-1/2016 (H5N8)	*PB2*	A/duck/India/10CA01/2016 (H5N8)	EPI858833	99.4
A/Cygnus atratus/Hubei/2Z2-O/2016(H5N8)	*PB1*	A/duck/India/10CA01/2016 (H5N8)	EPI858834	99.6
	*PA*	A/Bar-headed Goose/Qinghai/BTY9-LU/2016 (H5N8)	EPI774258	99.6
	*HA*	A/turkey/Poland/83/2016 (H5N8)	EPI869931	99.5
	*NP*	A/common tern /Uvs-Nuur Lake/26/2016 (H5N8)	EPI836615	99.6
	*NA*	A/duck/India/10CA01/2016 (H5N8)	EPI858838	99.6
	*MP*	A/duck/India/10CA01/2016 (H5N8)	EPI858840	99.5
	*NS*	A/duck/India/10CA01/2016 (H5N8)	EPI858839	99.8

**Fig. 1:**
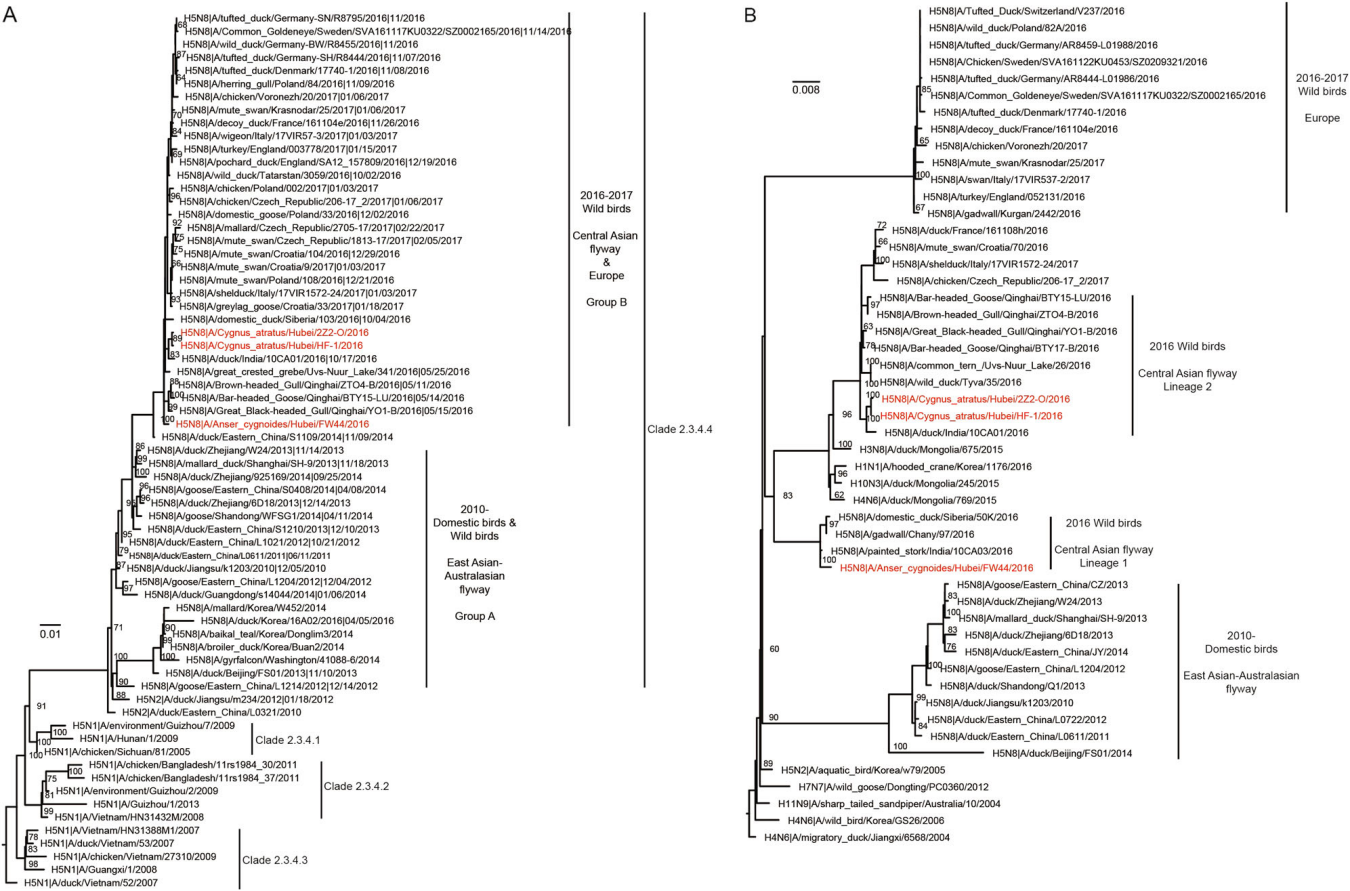
Maximum-likelihood phylogenetic tree for the HA (1704 nucleotides) and PA (2151 nucleotides) genes of H5N8 influenza viruses. The trees were rooted with the earliest strain among the selected sequences. The isolated H5N8 strains in this study are coloured in rose. Node labels indicate bootstrap values, and bootstrap values greater than 60 are shown

The H5N8 HA phylogenetic tree revealed that the *HA* genes of all three H5N8 viruses isolated in this study belonged to clade 2.3.4.4 group B^3, 19^, clustered with 2016–2017 H5N8 viruses from wild birds in the Central Asian flyway and Europe, and formed a separate branch together (Fig. 1a). The three H5N8 viruses were likely derived from the H5N8 domestic poultry circulating in eastern China in 2014; however, the evolutionary history is unclear. The two isolates from black swans shared a common ancestor with A/duck/India/10CA01/2016 (isolated on October 17, 2016), and FW44 likely shared a common ancestor with Qinghai-lake H5N8 viruses.

The PA and NP genes of the three viruses clustered with viruses from Mongolia, Siberia, India, and Qinghai Lake, located in the Central Asian flyway (Fig. 1b, NP tree not shown). This cluster was distinguished from those of H5N8 viruses circulating in 2010–2014 domestic birds in East Asia and 2016–2017 wild birds in Europe. Surprisingly, the three viruses were derived from two distinct lineages (Central Asian flyway Lineage 1 and Central Asian flyway Lineage 2), indicating that additional genetic reassortment occurred (Fig. 1b). These findings showed that FW44 virus was a novel reassortant in wild birds and clustered with viruses isolated in India, Siberia, and Chany.

### Molecular dating of H5N8 viruses in Asia

**Fig. 2:**
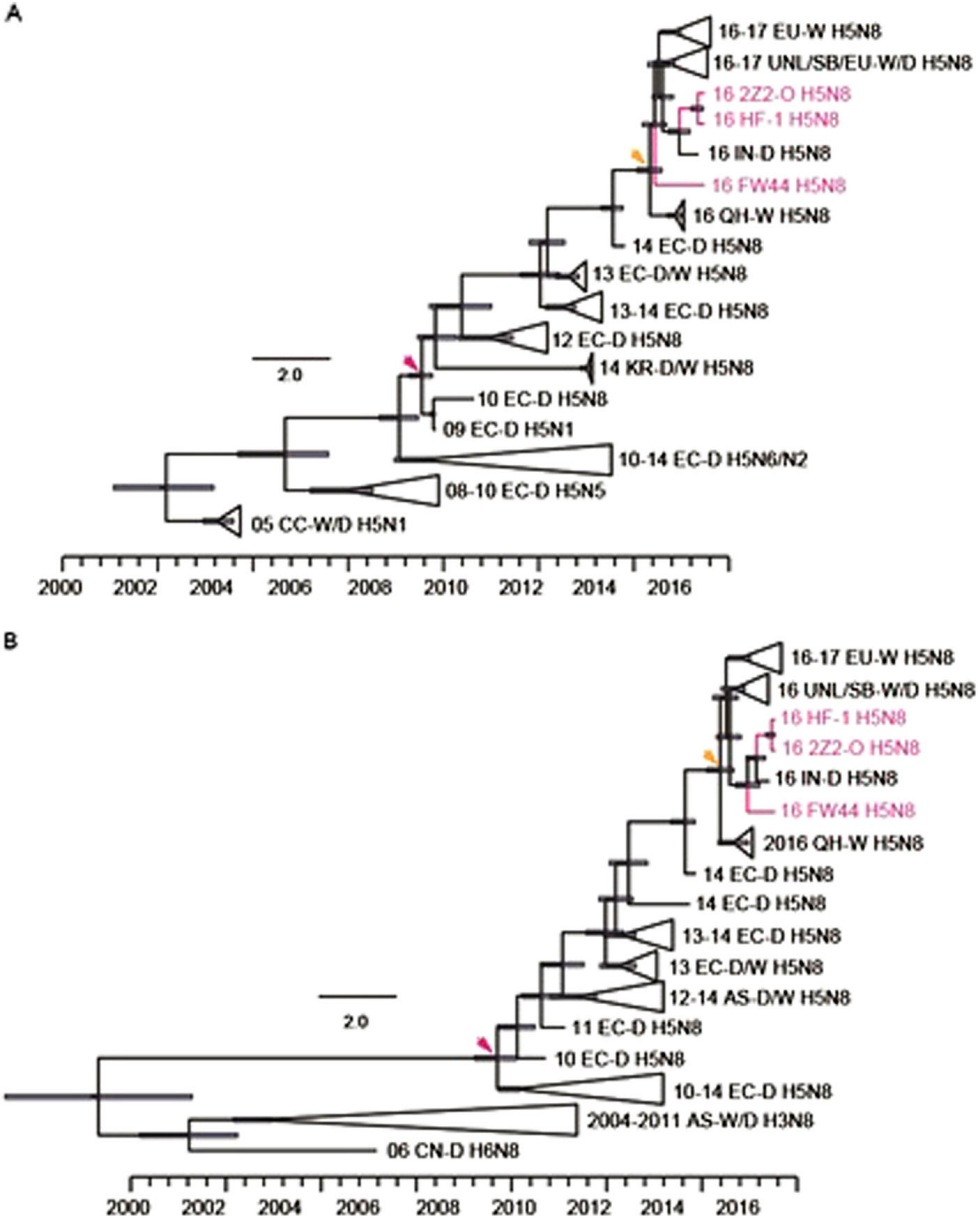
Maximum clade credibility (MCC) trees of hemagglutinin (HA; 1704 nucleotides) and neuraminidase (NA; 1395 nucleotides) genes. The strains in this study are indicated in rose, and the time of the most recent common ancestor (MRCA) of H5 and N8 is indicated with a rose arrow. The MRCA of Central Asian and European viruses is indicated with a yellow arrow. D domestic birds, W wild birds, QH Qinghai Lake, CN China, EC Eastern China, CC Central China, KR Korea, EU Europe, AS Asia, IN India, UNL Uvs-Nuur Lake, SB Siberia

The time at which H5 paired with N8 to generate H5N8 was estimated by molecular dating. Our analysis revealed that H5 was derived from domestic poultry HPAI H5Ny in August 2009 (95% highest posterior density (April 2009, December 2009)) (Fig. 2a). N8 likely originated from HxN8 viruses (mainly H3N8) circulating in wild and domestic birds in Asia in September 2009 (January 2009, March 2010) (Fig. 2b). This result indicated that the reassortment of HA and NA for H5N8 occurred in the short term in 2009 and probably occurred between domestic and wild aquatic birds.

The first H5N8 (A/duck/Jiangsu/k1203/2010) emerged in eastern China and belonged to the East Asian-Australasian flyway. Thus, we next estimated the induction time of H5N8 HA and NA into Central Asia and Europe by molecular dating (Fig. 2). The most recent common ancestors of H5N8 HA and NA emerged in Central Asia in July 2015 ((March 2015, October 2015)) (Fig. 2a) and July 2015 ([January 2015, October 2015]) (Fig. 2b), respectively. Molecular dating also indicated that the strains isolated in this study shared a common ancestor with those in Central Asia.

### Molecular markers of replication, virulence, and transmission determinants

HA cleavage sites of three viruses possessing multiple basic amino acids were confirmed, implying that the viruses were highly pathogenic in chickens (Table 2). The receptor-binding site at the 226–228 (H3 numbering) motif (QRG) suggested an avian-like (α 2-3-SA) receptor-binding preference^20^. However, the T160A mutation in the HA protein in the three H5N8 strains suggested that the binding affinity to human-like (α 2-6-SA) receptor may be increased^21, 22^. Two amino acid deletions in the NA stalk region (positions 58–59) were observed; these mutations may increase virulence in mammals^23^. Additionally, E627 and D701 in PB2 demonstrated that the isolates were not mammal-adapted avian influenza viruses. S31 in M2 and H274 in NA illustrated that the viruses were sensitive to M2 channel inhibitors and NA inhibitors, respectively (Table 2)^24, 25^.

**Table 2:** Characterisation of selected molecular markers of H5N8 viruses

Viruses (H5N8)	HA^a^ (H3 numbering)					NA^b^		PB2		M2
	Connecting peptide	160	226	227	228	274	Stalk deletion	627	701	31
FW44	REKRRKR	A	Q	R	G	H	58–59	E	D	S
2Z2-O	REKRRKR	A	Q	R	G	H	58–59	E	D	S
HF-1	REKRRKR	A	Q	R	G	H	58–59	E	D	S

*FW44* A/Anser cygnoides/Hubei/FW44/2016(H5N8), *2Z2-O* A/Cygnus atratus/Hubei/2Z2-O/2016(H5N8), *HF-1* A/Cygnus atratus/Hubei/HF-1/2017(H5N8)

^a^ H3 numbering

^b^ N2 numbering

### HA and NA genetic markers of virus host jumps

**Fig. 3:**
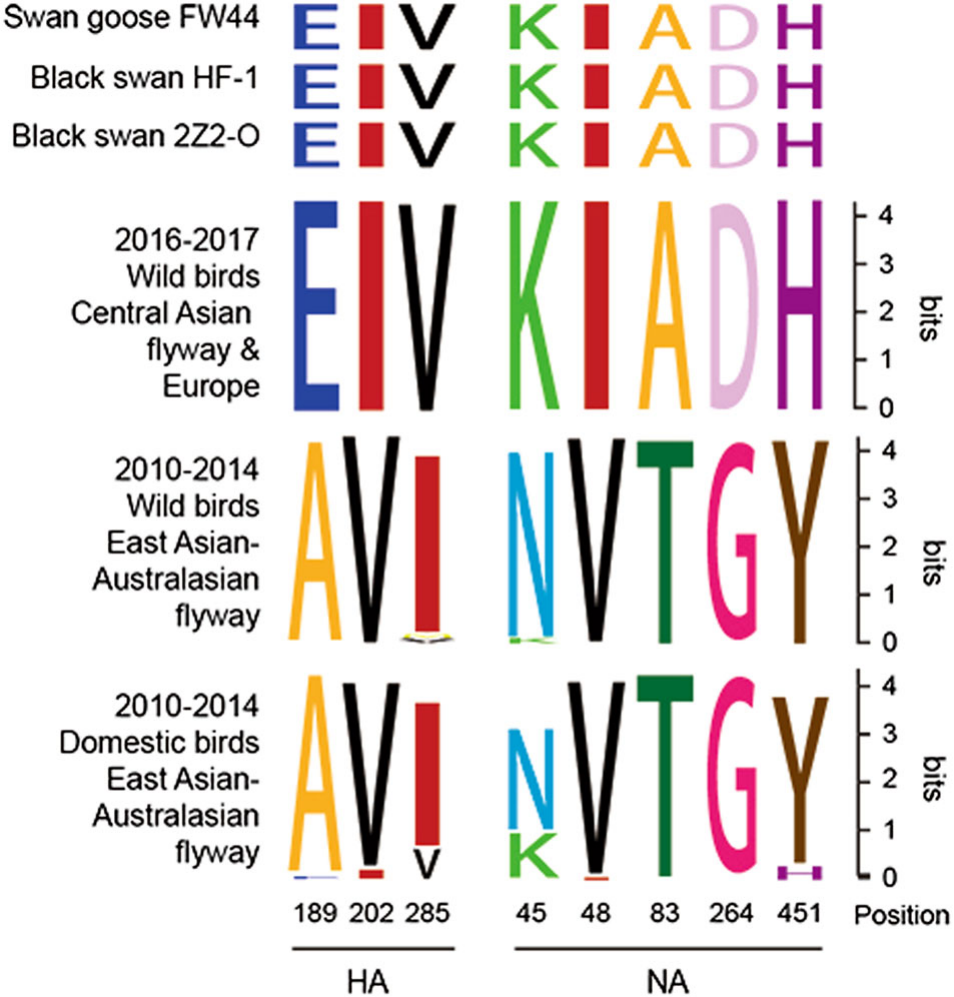
SeqLogo analysis of amino acid substitutions in H5N8 viruses. HA and NA (H3 and N2 numbering) are shown

Circulation of H5N8 from domestic poultry to wild birds suggested host adaptation. Therefore, we next compared the amino acid sequences of HA and NA in available H5N8 viruses from domestic poultry and wild birds in the public database Influenza Research Database (https://www.fludb.org/brc/home.spg?decorator=influenza) and Global Initiative on Sharing All Influenza Data (http://platform.gisaid.org/epi3/frontend; Fig. 3). In particular, eight amino acids (three in HA, five in NA) changed gradually during the adaption process from domestic poultry to wild birds. Amino acid sites at positions 189, 202, and 285 of HA protein (H3 numbering) in 2010–2014 H5N8 in domestic birds were A/E (89/2), V/I (86/5), and I/V (80/11); those in 2010–2014 wild birds were A/E (69/1), V, and I/M/V (66/2/2); and those in 2016–2017 H5N8 in wild birds were E, I, and V. The mutations (A189E, V202I) were within or adjacent to the HA 190 helix and 220 loops^26^. Similarly, amino acid sites at positions 45, 48, 83, 264, and 451 of NA protein (N2 numbering) in 2010–2014 H5N8 in domestic poultry were N/K (34/15), V/I (48/1), T, G, and Y/H (45/4); those in 2010–2014 wild birds were N/K (51/2), V, T, G, and Y; and those in 2016–2017 H5N8 in wild birds were K, I, A, D, and H. Thus, these above substitutions occurred during virus transmission from domestic birds to wild birds. The three isolates in this study all possessed the featured amino acids of wild bird viruses (Fig. 3).

## Discussion

Using routine virus isolation and subtype identification, we determined the occurrence of H5N8 avian influenza virus in migrating birds in lakes, wetlands, and LPMs in Central China since 2015. No H5N8 virus was isolated in LPMs in regular sampling, and only three H5N8 viruses were isolated from dead wild birds, additionally, in spring 2016, massive wild birds died for H5N8 virus infection almost at the same time worldwide^3, 4^, suggesting that H5N8 disseminated mainly through migratory birds. Moreover, we demonstrated there was a novel reassortant H5N8 virus (FW44) containing genes similar to those of Qinghai-H5N8 viruses, except NP and PA. The complete genomes of two black swan isolates were similar to those of Qinghai-H5N8 viruses.

Phylogenetic analyses supported that continuous reassortment occurred in H5N8. Besides the two known reassortments, Qinghai Lake and Central Europe, we also isolated a third reassortant FW44, in which PA and NP genes were similar to those of viruses in domestic and wild birds from India and Siberia, whereas other genes were similar to those of Qinghai-H5N8 viruses. Furthermore, Central European H5N8 isolates, such as A/tufted duck/Germany/AR8444/2016, were clearly distinguished from the Russia-Mongolia lineage (Qinghai lineage) due to the presence of two new segments (PA and NP) from local domestic and wild birds^27^. Notably, PA and NP genes in Germany isolates and viruses in this study belonged to different lineages, suggesting that the H5N8 virus tended to alter genes related to viral replication.

From our analysis of amino acid variations in HA and NA, we found that eight sites could be related to “host-jump” from domestic to wild aquatic birds. Previous studies have shown that each HA monomer contains a number of conserved amino acids surrounded by three conserved secondary structures, the 130-loop, 220-loop, and 190-α-helix, at the receptor-binding site^28^. Notably, the HA189 and HA202 mutations are in close proximity to the receptor binding site, which may affect the receptor binding preference. In addition, NA264 is adjacent to the active site of NA (Glu276), which is associated with sensitivity to NA inhibitors^28^. Importantly, all of the sites reported in this study were novel and have not been reported previously.

**Fig. 4:**
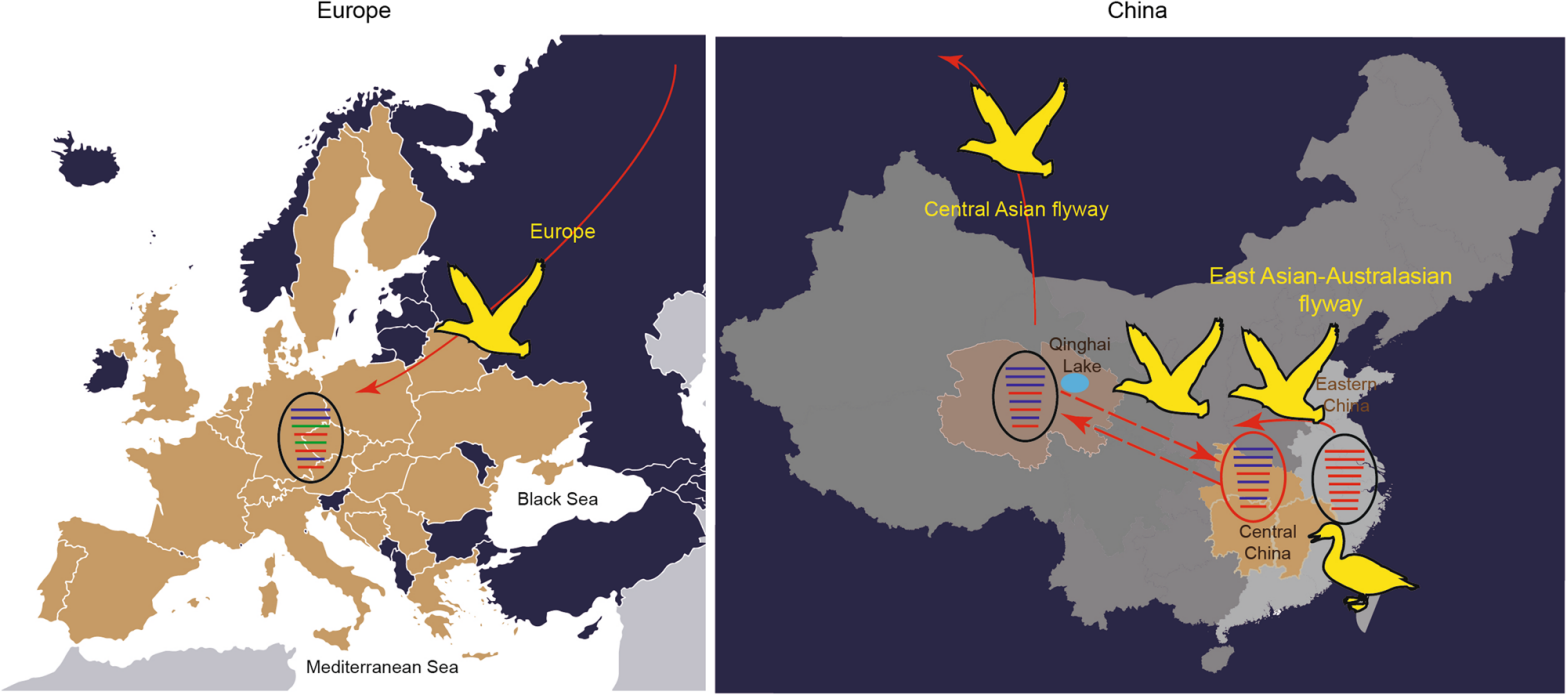
The proposed reassortment and transmission model of H5N8 virus among the East Asian-Australasian flyway, Central Asian flyway, and Europe. Eastern China, Central China, and Qinghai Lake are coloured in light grey, light orange, and bright green, respectively. The countries in which H5N8 outbreaks occurred in Europe are coloured in light brown. Transmission routes for H5N8 virus are indicated with a red solid arrow, and the probable transmission route between Qinghai and Central China is indicated with a red dotted arrow. Gene segments are coloured according to their origins. Notably, the PA and NP segments of H5N8 viruses in Europe were from unidentified viruses and are coloured in green

Our data suggest that wetland ecosystems in Central China play an important intermediate role in connecting avian influenza viruses carried by migratory birds between the Central Asian flyway and East Asian-Australasian flyway. The viruses in this study were isolated from wild birds in the East Asian-Australasian flyway^29, 30^, but the genetic features were closely related to H5N8 viruses along the Central Asian flyway. The proposed diagram suggested that the wild birds carried H5N8 viruses from Eastern China to Central China and then to the Qinghai Lake, where migratory birds fly north and bring the viruses into Mongolia, Russia, and Europe. During virus dissemination, the internal gene segments of viruses from domestic ducks were replaced by those circulating in wild birds, except for the NS gene (Fig. 4). Our analysis indicates that wetlands and lakes may be important intermediate spots connecting the East Asian-Australasian and Central Asian flyways, allowing H5N8 viruses to spread.

Unlike avian influenza viruses in domestic poultry, in which epidemics can be contained by culling, the migration of birds is uncontrollable. H5N8 virus infections in wild birds were widely reported during 2016/2017 winter. Our data described amino acid mutations during cross-species transmission; however, further studies are needed to determine whether these substitutions correlate with host adaptation. Our study also emphasised the need for continual surveillance of wild birds and LPMs for avian influenza virus.
